# The effect of Universal Teacher–Child Interaction Training on Hispanic teachers’ sense of self-efficacy in early childhood education and care settings

**DOI:** 10.1186/s40723-023-00115-6

**Published:** 2023-04-13

**Authors:** Alexandra Rivas, Angela Mooss, Christine Hughes Pontier, Jackie Romillo, Emma Muñoz

**Affiliations:** 1 Early Childhood Development Citrus Health, Citrus Health Network Inc., Miami, FL USA; 2 Behavioral Science Research Institute, Miami, FL USA

**Keywords:** Teacher–Child Interaction Training (TCIT), Inservice training, Teacher Sense of Self-efficacy, Hispanic, Early childhood education and care (ECEC)

## Abstract

Disparities exist in the availability of high-quality early childhood education and care settings (ECEC) across communities within the United States. Teachers have an imperative role in fostering children’s socioemotional development; however, when the classroom climate deteriorates due to disruptive behavior, meeting these emotional and learning needs becomes more difficult. Dealing with challenging behaviors can lead to emotional exhaustion which is directly linked to a decrease in teacher sense of efficacy. Teacher–Child Interaction Training-Universal (TCIT-U) targets teachers’ skills to provide quality interactions and decrease child behavior problems. Despite evidence that teacher sense of self-efficacy can inhibit negative teaching practices, a lack of research has explored this construct as related to TCIT-U. The current study is a randomized, wait-list control study measuring the change of teachers’ sense of self-efficacy after participating in TCIT-U, and the first known of its kind. The study included mostly Hispanic (96.4%) teachers (*N* = 84) of ECEC programs across 13 unique sites serving 900 children ages 2–5 years from low-income, urban areas. Results from inferential statistics and hierarchical linear regression tests demonstrated TCIT-U as an effective intervention to improve teachers’ sense of efficacy in classroom management, instructional strategies, and student engagement. In addition, this study contributes to the effectiveness of TCIT-U as an in-service training which targets teacher communication skills for teachers with diverse backgrounds in ECEC settings with mostly dual language learners.

## Introduction

Over half of children between the ages of 2 and 5 years regularly attend early childhood education and care settings (ECEC) before entering kindergarten (National Academies of Sciences, Engineering, & Medicine, 2018). High quality ECEC is imperative for a child’s cognitive, social, and emotional development. A meta-analysis conducted by Mccoy and colleagues (2017) found that participation in ECEC had positive long-term effects including significant reductions in special education placement, grade retention, and improved rates of high school graduation. As children transition from home to ECEC settings, the role of the teacher is not only impactful in the context of a child’s cognitive development, but also on their socioemotional functioning (Buettner et al., 2016; Cruby et al., 2013; Egert et al., 2020; Plotka, 2019; Zhang & Sun, 2011). Thus, it is more important than ever to implement training that supports teachers in their positive interactions with children, while boosting teacher’s sense of self efficacy to address challenging issues that arise. Teacher–Child Interaction Training-Universal (TCIT-U) was designed with the aim of “promoting a positive classroom environment, preventing future child behavior problems, addressing current externalizing issues, and decreasing teacher burnout” (Gershenson et al., 2010, pg. 281). TCIT-U was adapted from Dr. Shelia Eyberg’s Parent–Child Interaction Therapy (PCIT), an evidence-based treatment for children ages 2–7 years (Funderburk & Eyberg, 2011). TCIT-U incorporates in-vivo coaching which instructs teachers to modify student behavior with attention, through modeling, and data-based feedback (Fernandez et al., 2014). Training incorporating components of in-vivo coaching have led to desirable outcomes for teachers (Noell et al., 2005; Reinke et al., 2008). The TCIT-U model also includes live coaching of teacher skills such as immediate positive feedback to reinforce teacher’s appropriate skill usage, opportunities to quickly correct errors so repetition of incorrect techniques is avoided, and skills adaptation so teachers can manage specific classroom behaviors as they arise. There is a dearth of studies specifically addressing the change and stability in self-efficacy among teachers after in-service training, specifically TCIT-U in ECEC. The present study contributes to the importance of implementing programs targeting high quality teacher–child interactions which increase teacher sense of self-efficacy.

### Teacher self-efficacy

Self-efficacy refers to an individual’s belief in their capability to perform actions to produce specific performance attainments (Bandura, 1997). Teacher self-efficacy refers to a teacher’s belief in his or her own capability to successfully accomplish a specific teaching task. A higher sense of self-efficacy enhances teachers’ abilities to respond effectively to stressful and challenging situations, (Bray-Clark & Bates, 2003) and deliver long-term, higher quality instruction (Holzerberger et al., 2013; Künsting et al., 2016; Lipscomb et al., 2022). Findings from a study analyzing questionnaires completed by ECEC, kindergarten, and first grade teachers indicated that teachers experiencing higher levels of stress spent less time teaching literacy and numeracy and interacting with parents. In contrast, teachers experiencing higher levels of efficacy spent increased time teaching both cognitive skills and social–emotional skills, and communicating with parents (Fantuzzo et al., 2012). In addition, teachers with higher self-efficacy are more willing to work with children who exhibit behavioral issues, rather than referring them to special education (Tschannen-Moran et al., 1998). Studies have also shown a significant interaction among teachers’ self-efficacy, classroom quality, and gains in school readiness (Williford et al., 2013), including language and literacy gains (Guo et al., 2010).

Professional development and in-service training can positively influence teacher self-efficacy (Bray-Clark & Bates, 2003; Santiago et al., 2022; Toran et al., 2017). For many educators who do not participate in formal preparation programs, in-service training often overlaps as default preparation for practice (Institute of Medicine & National Research Council, 2015). Training that target interactions between teachers and children within ECEC settings are among the most promising intervention for supporting children’s positive social and emotional development and academic competencies. Training that bolsters teacher sense of self-efficacy is critical in supporting greater work engagement (Lipscomb et al., 2022), in addition to establishing and maintaining positive teacher–child interactions within early education settings (VanLone et al., 2022).

### Teacher–child interactions

Young children who have positive teacher–child relationships characterized by warmth, affection, and open communication (Ferreira et al., 2016) tend to exhibit fewer internalizing and externalizing problems (Baardstu et al., 2022; Silver et al., 2005; Zhang & Sun, 2011). Considering the importance of an ECEC educator’s role, research shows that teachers consistently feel they do not have sufficient training to meet both the emotional and learning needs of young children (Gebbie et al., 2012; Humphries et al., 2018; Reinke et al., 2011).

A young child’s behavioral outcomes are partially dependent on how adults, like teachers, respond to the child's behavior. Hallmarks of disruptive behavior in children include inattention, hyperactivity or displays of oppositionality to the degree that the learning environment is negatively affected (Gebbie, et al., 2012; Yoder & Williford, 2019). Teachers may resort to reactive and punitive responses, which do not allow for children to learn self-regulation, and which may contribute to a self-sustaining cycle of classroom disruption (Jennings & Greenberg, 2009). Negative interactions have been shown to be ineffective at helping children regulate emotions and control behavior, thus contributing negatively to a teacher’s well-being, and sense of self-efficacy (Plotka, 2019; Spilt et al., 2011). Teachers’ levels of self-efficacy are predictive of their effectiveness in managing children’s behavior (Perren, 2017; Plotka, 2019; Santiago et al., 2022).

### Disparities in ECEC settings

Disparities exist in the availability of high-quality early childcare and education across communities (Bassok et al., 2016; NASEM, 2018; Valentino, 2017). Socioeconomic status is one risk factor that has been associated with externalizing behavior by limiting access to resources that promote healthy child adjustment (Bradley & Corwyn, 2002). Moreover, children with externalizing problems are more vulnerable when it comes to developing positive teacher relationships (Baardstu et al., 2022; Henricsson & Rydell, 2004). High-quality early childhood education is important in reducing the initial achievement gap for children coming from lower socioeconomic backgrounds and for positively impacting children’s future academic and emotional well-being (Aguiar et al., 2020; McCoy et al., 2017; Reardon & Portilla, 2016). Beyond academic gains, the positive impacts on social emotional development from high-quality teacher–child interactions (Liew, 2012) are imperative for Hispanic youth, as they are less likely to receive mental health treatment compared with their White, Non-Hispanic peers (Caballero et al., 2017; Dettlaff & Cardoso, 2010; Yoshikawa et al., 2013). ECEC teachers with a greater sense of self-efficacy are better prepared to meet the emotional and academic learning needs of young children.

Research that supports that TCIT-U has led to improved teacher skills and reduced behavioral concerns for children as perceived by teachers (Garbacz et al., 2014; Lyon. et al., 2009). Higher teacher sense of self-efficacy is linked to improvements in classroom climate, commitment to the profession, prevention of burnout, and willingness to implement new teaching practices (Jennings & Greenberg, 2009; Klassen & Chiu, 2010; Lipscomb, et al., 2022; Rimm-Kaufman & Sawyer, 2004; Ware & Kitsantas, 2007). Given the significant role that ECEC teachers have in facilitating the socioemotional skills needed for school readiness, and to minimize disparities for children at higher risk of negative developmental outcomes, teachers should receive in-service training which improves their self-efficacy.

### Current study

The current study was a wait-list control, randomized design which implemented the TCIT-U program. This study was conducted by a Federally Qualified Health Center (FQHC) in Miami-Dade County, which received funding from the county’s dedicated source of revenue established by voter referendum to improve the lives of all children and families in Miami-Dade County. Most of the children and families served by the Florida childcare subsidy are low-income and from underserved areas of Miami-Dade. Teacher participants (*N* = 84) of the TCIT-U training were primarily Hispanic (96.4%) and all female, with varying levels of professional experience across multiple ECEC sites supported by the early childhood development services provided by the Federally Qualified Health Center. The aim of the current study was to answer the following research questions:

- To what extent does TCIT-U significantly change Hispanic teachers’ sense of self-efficacy compared to the control group who did not receive the training?
- To what extent do changes in self-efficacy persist after the intervention?
- To what extent does TCIT-U predict changes in self-efficacy above and beyond other confounding factors, such as teacher experience, education, and structural factors of the classroom?

### Research design

**Fig. 1:**
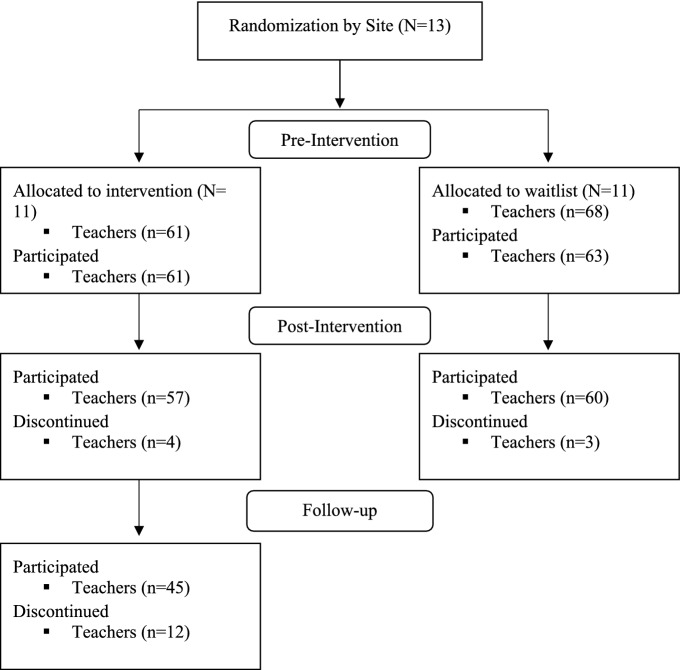
Multi-site assignment

The present study included random assignment of ECEC sites to either an intervention group, which received TCIT-U, or a delayed intervention group which served as the comparison, or treatment as usual (TAU) group. A priori power analysis was conducted to determine the minimum sample size required to test the study hypotheses. Results indicated the required sample size required to achieve 80% power for detecting a medium effect, at a significance criterion of *α* = 0.05, was *N* = 15 for a paired-samples *t* test and a sample of *N* = 44 for a multiple regression. Thus, the obtained sample size of 55 paired samples and 84 participants was adequate to test the study hypotheses. Nearly all teachers and sites in the delayed intervention group later received the TCIT-U intervention the following academic semester except for one site which was challenged with scheduling during the COVID-19 pandemic. The study was grant funded and a research protocol was submitted to the Western Institutional Review Board (WIRB) for review. WIRB’s IRB Affairs Department reviewed the exemption criteria under 45 CFR §46.101(b) (1) and determined the project to be exempt from IRB oversight as: Research conducted in established or commonly accepted educational settings, involving normal educational practices, such as (i) research on regular and special education instructional strategies, or (ii) research on the effectiveness of or the comparison among instructional techniques, curricula, or classroom management methods. The following figure (Fig. 1) depicted the multi-site (*N* = 13) assignment over two academic years, including fall and spring terms, and the total number of teachers allocated to intervention and waitlist control. Only sites that received the intervention had follow-up assessments, Fig. 1.

### Participants

The TCIT-U program included four coaches from the Federally Qualified Health Center, who were early childhood mental health and child development specialists, including Licensed Clinical Social Workers and certified Parent Child Interaction Training trainers. The coaches that worked directly with teachers were bilingual English and Spanish speakers. Developers of TCIT-U trained the coaches to provide training and live coaching for teachers in multiple child-care settings across Miami-Dade over the course of 14 weeks. The characteristics of teachers that participated in the study are described in Table 1.

**Table 1:** Teacher participant descriptive information

	*N* = 84	%
Gender		
Female	84	100%
Male	0	0%
Race/Ethnicity		
Hispanic/Latino	81	96.43%
Black/African American	2	2.33%
Non-hispanic white	3	4.65%
Mean Age *(SD)*	43.38 (13.11)	
Education		
HS graduate or GED	26	30.20%
Some college	14	16.30%
Associate degree	15	17.40%
B.A. degree	25	29.10%
Higher degree (M.A.)	4	4.70%
Proficiency in english		
Yes	36	41.86%
No	38	44.19%
Mean years teaching experience *(SD)*	11.12 (*8.53*)	
Mean years at current site *(SD)*	4.65 (*5.32*)	

All teachers were female (*N* = 84), and nearly all identified as Hispanic/Latino (96.4%). Approximately one-third of teachers had a bachelor’s degree or higher and 33.7% reported having had some college courses or an associate degree. Teachers had an average of 11.1 years of teaching experience and an average of 4.6 years at their current site. A total of 963 children, ages 2–5 years, with an average age of 3.9 years participated in the TCIT-U evaluation. The intervention did not specifically target children with behavioral disorders or disruptive behaviors, and all sites served a universal population which included children with special needs.

### Measures

#### Teacher sense of efficacy scale (TSES)

The Teacher Sense of Efficacy scale (Tschannen-Moran & Hoy, 2001) is a 24-item instrument exploring teacher efficacy through the following domains: Student Engagement (SE), Instructional Strategies (IS), and Classroom Management (CM). Student engagement reflects emotional engagement (interest and enthusiasm) and behavioral engagement (participation) in school (Van Uden et al., 2013). The efficacy for instructional strategies includes a teacher’s confidence in designing and implementing classroom activities, instructional styles, and assessment according to the needs of individual students (Wolters & Daugherty, 2007). Manning and Bucher (2013) describe classroom management as all strategies that ensure physical and psychological security in the classroom, and all techniques designed to regulate student behavior and build self-discipline. The survey has a 9-point response scale, where a higher response on the scale indicated greater self-efficacy. Tschannen-Moran and Hoy conducted a second-order factor analysis yielding results that supported the overall reliability of 0.94 for the 24-item scale TSES tool. Heneman and colleagues (2006) conducted a study using confirmatory factor analysis and found that reliability for the subscales and total scores were high, ranging from 0.75 to 0.90. In addition, the factor structure was consistent for teachers across all grade levels, demonstrating the generalizability of TSES. Composite scores for each subscale (SE, IS, and CM) were created by averaging items that load on each factor. A certified translator translated the TSES tool into Spanish and reviewed the tool to detect semantic and/or conceptual differences between the original and translated version. The translated version was used among teachers whose preferred language was Spanish.

### Procedure

Developers of TCIT-U trained coaches in an initial 4 day workshop, provided two onsite consultation visits, and monthly telephone consultations across the academic year. The coaches then supported the teachers’ use of TCIT-U skills in real time in the classroom approximately once a week for 1 h over 6 weeks. The training occurred in two phases, Child-directed Interaction Training (CDI) and Teacher-directed Interaction Training (TDI). Following the first year of training, coaches decided to implement a booster session 13 weeks after completion of training.

#### CDI training

The first phase of the training is Child-directed Interaction Training, which focuses on promoting positive and differential social attention to improve teacher–child relationship. Following the Parent–Child Interaction Therapy (PCIT) model, teachers implement PRIDE, a mnemonic device used to: *Praise* appropriate behavior, *Reflect* on appropriate child speech, *Imitate* appropriate child behavior, *Describe* appropriate behavior, and *Exhibit* enthusiasm when interacting with children. Teachers also learned to refrain from negative talk and unnecessary direct commands. Skills taught in the CDI phase continued into the second phase of the training, Teacher-directed Interaction training.

#### TDI training

During the TDI phase teachers learned how to deliver clear, direct commands to reward child compliance and utilize effective strategies for child noncompliance. The primary goal of the second phase of TCIT-U is to change ineffective teacher–child interaction patterns. The second phase also includes classroom management strategies that further reduce behavioral problems.

#### Training sequence

For CDI, coaches provided 3 days of training, followed by 6 weeks of coaching teachers, with a midway feedback session. For TDI, training coaches provided 3 days of training, followed by 6 weeks of coaching teachers, with a midway feedback session. There was a total of approximately 12 h of training and 24 h of coaching for teachers. Individual coaching sessions occurred during a variety of typical classroom situations including one-on-one lessons with an individual child, small group activities, or whole class instruction. TCIT-U Coaches code teachers’ skill used to assess progress in both CDI and TDI phases. TCIT-U Coaches provided immediate verbal feedback and support as the teacher interacted with children. During coaching, TCIT-U Coaches used specific praise to identify and reinforce teachers’ use of skills as situations occurred and prompted teachers to use a specific skill during select opportunities. Immediately following coaching sessions, TCIT-U Coaches debriefed with teachers and provided guidance on problem-solving with individual children exhibiting disruptive behavior when necessary.

A teacher information form was administered at the beginning of each semester to capture teachers’ total experience, level of education, and demographic information. Teachers were not compensated for participating in the training and were informed that participation was voluntary. Teachers who agreed to participate in the study were provided with written informed consent. Teachers also completed the Teacher’s Sense of Efficacy Scale (Tschannen-Moran & Hoy, 2001), 2 weeks before their baseline initial training, after the second phase TDI training, and once more at follow-up (6–8 weeks after TCIT-U post-intervention training). Only teachers who had received the TCIT-U intervention completed the follow-up TSES assessment.

## Results

Average composite scores were created for each subscale at baseline, post-intervention and following the intervention (Table 2). To examine if teacher factors (level of education, current length of experience at current site, total teaching experience, proficiency in English, and age) or extraneous factors (spring or fall term, first/second year, and number of children in each class) were associated with teachers’ sense of efficacy ratings, a two tailed Kendall’s-tau b correlational test was used. Neither teacher factors nor other extraneous factors were statistically significantly associated with teacher sense of self-efficacy ratings (*p* > 0.05). Twelve teachers (14.3%) completed baseline assessments but were lost at follow-up. These teachers were not significantly different from those with complete data across intervention and treatment as usual conditions (6.6% v 11.8%) *χ*^2^ (1) = 0.090, *p* = 0.309. Self-efficacy scores were not significantly different between teachers who were lost to follow-up and teachers without missing data, *p* > 0.05. Only current teaching experience at specific sites was statistically different between teachers lost to follow-up and teachers without missing data, *U* = 372, *z* = − 2.682, *p* = 0.007*.* Teachers with missing data had significantly less current experience (mean rank = 37.50) than teachers without missing data (mean rank = 66.77).

**Table 2:** *T* test results of teachers’ sense of efficacy in the intervention group and treatment as usual groups at baseline and post-intervention

	Treatment group	Timepoint	*N*	*X*	SD	*df*	*t*	*P*
Efficacy in student engagement	Experimental	Baseline	57	7.29	0.57	56	5.48	< 0.001
		Post	57	7.98	1.02			
	TAU	Baseline	55	7.65	0.98	54	− 1.03	0.31
		Post	55	7.58	0.99			
Efficacy in instructional strategies	Experimental	Baseline	57	7.32	0.57	56	5.98	< 0.001
		Post	57	8.10	1.03			
	TAU	Baseline	55	7.55	1.06	54	− 0.14	0.89
		Post	55	7.54	1.01			
Efficacy in classroom management	Experimental	Baseline	57	7.21	0.58	56	7.75	< 0.001
		Post	57	8.20	1.05			
	TAU	Baseline	55	7.48	0.97	54	0.97	0.34
		Post	55	7.55	0.97			

### ***Effects of the TCIT-Uintervention***

TSES scores met the assumption of normality, and paired sample *t* tests were used to determine if changes in teacher sense of efficacy significantly improved from baseline to post-intervention for teachers in the intervention group and for teachers in the control group (Table 2).

For teachers who received TCIT-U training, there were significant improvements for the following domains: instructional strategy, classroom management, and student engagement for teachers who received the training (*p* < 0.001). The greatest improvement in self-efficacy was seen for the subscale classroom management. There was a significant increase in classroom management efficacy (*M* = 8.20, SD = 1.05) following the training compared to before the training (*M* = 7.21, SD = 0.97), *t* (56) = 7.748, *p* < 0.001. Cohen’s d (1998) was used to assess effect size for changes in scores from baseline to post-intervention and the intervention had a large effect (*d* = 0.95–99) across measures. Changes in self-efficacy for teachers in the treatment as usual group was not statistically significant from baseline to post-intervention (*p* > 0.05).

A one-way repeated measures ANOVA was conducted to determine whether there were statistically significant differences in TSES measures over the course of the intervention for participating teachers. The assumption of sphericity was violated, as assessed by Mauchly’s test of sphericity, *χ*^2^ (2) = 50.579, *p* < 0.05. Therefore, a Greenhouse–Geisser correction was applied (*ε* = 0.624). TCIT-U elicited statistically significant changes for TSES measures for teachers in the intervention group over time, F (1.249, 69.942) = 30.810, *p* < 0.001. Post hoc analysis with a Bonferroni adjustment revealed that changes in Instructional Strategies statistically increased from pre-intervention to post-intervention (0.716(95% CI 0.334, 1.098, *p* < 0.001) and from post-intervention to follow-up (0.263(95% CI 0.120, 0.405, *p* < 0.001); Classroom Management statistically increased from pre-intervention to post-intervention (0.927(95% CI 0.561, 1.292, *p* < 0.001), but not from post-intervention to follow-up (0.139(95% CI 0.000, 0.279, *p* = 0.051); Student Engagement statistically increased from intake to post-intervention (0.797(95% CI 0.397, 1.196, *p* < 0.001) and from post-intervention to follow-up (0.184(CI 0.004, 0.364, *p* < 0.05).

An ANCOVA test was used to examine the effect of the treatment condition on the collective instructional strategies, classroom management and student engagement post-intervention scores, controlling for baseline scores. After adjustment for pre-intervention TSES measures, there was a statistically significant difference in post-intervention TSES scores between teachers who received the TCIT-U intervention and teachers in the control condition, F (1, 109) = 40.190, *p* < 001, η2 = 0.269. Overall average TSES scores were greater in the intervention group (*M* = 8.09, SD = 0.53) compared to the control group (*M* = 7.55, SD = 0.97).

## Discussion

Numerous studies have demonstrated that Teacher–Child Interaction Training has promising success for improving teacher–child interactions and teacher’s effective behavior management skills (Budd et al., 2016; Davidson et al., 2020; Fawley et al., 2020; Fernandez et al., 2015; Fernandez et al., 2015; Garbacz et al., 2014; Lyon et al., 2009; Santiago et al., 2022). To the authors’ knowledge, this was the first quasi-experimental study that explored how TCIT-U affects teachers’ sense of self-efficacy among a predominately Hispanic (96.4%) teacher population in the United States. Studies identifying effective ways to enhance teacher self-efficacy have significant implications for promoting positive children’s development and minimizing disparities in academic learning. A prior study demonstrated that TCIT-U improved teacher skills and reduced problem behaviors of preschool children who are English language learners in a rural public-school setting (Fawley et al., 2020). The current study advances the evidence of the effectiveness of TCIT-U with teachers with varying levels of English proficiency, and with children who are dual English language learners. The current study provided coaching in teachers’ preferred language, English, or Spanish. Teachers also spoke to children in both English and Spanish. All sites were located in predominantly Hispanic, urban areas, and served children participating in the Florida School Readiness Program which provides low-income families financial support for early child education and care. Teacher–Child Interaction Training can improve the quality of early childhood education, which is integral to reducing the initial achievement gap and positively impacting future academic and emotional well-being for children coming from lower socioeconomic backgrounds (Aguiar & Aguiar, 2020; Duncan & Sojourner, 2013; McCoy et al. 2017).

The current study included teachers with varying levels of educational degrees and range of teaching experience. No statistically significant associations were found between educational degree, years of teaching experience and teachers’ sense of self efficacy. This finding highlights the program’s effectiveness across both novice and experienced teachers as well as teachers with varying educational degrees. Past research has shown mixed effects regarding these teacher characteristics. Some studies have found positive associations between teachers’ educational background and higher observed quality of educational practice in preschool classrooms (Guo et al., 2011). However, in a review of seven large scale studies, Early and colleagues (2007) found contradictory effects of formal teacher education on quality of ECEC settings. Regarding teacher experience, Guo and team (2011) found that teachers’ teaching experience was not a significant predictor of teachers’ self-efficacy, while Toran (2017) found a significant relationship between the duration of professional experience and teacher self-efficacy in a study which included 191 preschool teachers. Moreover, consistent with previous research, the current study did not find that the number of children in each classroom was associated with teacher sense of self-efficacy (Toran, 2017).

The current study sought to establish the impact of TCIT-U on teachers’ sense of self efficacy in classroom management, instructional strategies, and student engagement. TCIT-U was found to significantly increase teachers’ sense of self-efficacy following the training, and several weeks beyond the training. Findings indicated that TCIT-U had a sustainable positive impact on teachers’ sense of self efficacy, which aligns with other findings on the long-term stability of self-efficacy beliefs within in-service teachers (Künsting et al., 2016). Classroom management was the area in which teachers gained the greatest sense of self-efficacy following the training. Classroom management can be described as strategies to encourage desirable behaviors through positive reinforcement and creating a less disruptive environment (Manning & Bucher, 2013). In addition, effective classroom management has been linked to children's greater behavioral and cognitive self-control and less time spent off-task in the classroom (Rimm-Kaufman et al., 2009). It is expected that efficacy in classroom engagement would improve the most, since TCIT-U primarily aims to increase positive interactions between teachers and children and decrease disruptive behavior.

An improved sense of self-efficacy for teachers can improve turnover in ECEC settings and instructional quality. A study using teacher efficacy scales with 26,257 teachers confirmed that teacher efficacy significantly predicted teacher professional commitment (Ware & Kitsantas, 2007). Another study using structural equation modeling to analyze questionnaire results from 610 teachers found that teacher efficacy in handling student misbehavior is a central feature in the relationship between perceptions of student misbehavior and emotional exhaustion. Emotional exhaustion was found to be positively related to teacher attrition (Tsouloupas, 2010). Furthermore, teachers who are emotionally exhausted may resort to excessively punitive responses which do not support children’s development of self-regulation and may sustain disruptive behavior. Given the implications of the impact of teachers’ sense of self-efficacy, there is a high need for interventions, such as TCIT-U, which improve teacher sense of self-efficacy through in-vivo coaching of universal classroom management strategies.

## Limitations

The current study did not incorporate booster sessions, which were coaching sessions to reinforce skills 3 months after the TCIT-U intervention, until the second year. Although, efficacy improvement from post-intervention to follow-up across year 1 and year 2 cohorts was overall significant, there was a loss to follow-up due to teacher turnover. We did not account for differences in efficacy notwithstanding booster sessions. Moreover, it would be advantageous to know if efficacy continues to improve or remains stable in the longer term. Although, we collected demographic data on teachers, we were unable to collect demographic data on children beyond age and sex. It would be beneficial to gain insight if the impact of the intervention on teachers’ sense of self-efficacy would vary based on differing teacher race/ethnicity and fluency in one language compared to the dominant race/ethnicity and primary language of children in their classroom. In addition, this study did not measure change in teachers’ skills from an outside observer. Future studies could investigate if teacher sense of self-efficacy mediated changes in teacher interaction skills, as measured by the Teacher Child Interaction Coding System tool after participating in TCIT-U. Moreover, future studies could measure teacher-observed child behavior change, after implementing TCIT-U to see if teachers with higher self-efficacy perceived that child problem behavior is reduced. Research in teacher self-efficacy utilizing TSES could be supported with qualitative interviews to better contextualize the findings. Findings from the current study cannot be generalized to all ECEC sites of teachers within Miami-Dade County. Although, TSES has been used globally and has been shown to be an effective measure across different cultures, there is limited studies on the psychometric properties in the context of its’ use with native Spanish speakers within the United States. Future studies could include confirmatory exploratory analysis to explore the validity of TSES with this specific population in both rural and urban areas.

## Conclusion

ECEC teachers play an important role in preventing behavior problems and promoting socioemotional health in the classroom. Universal, in-service training programs, such as TCIT-U which focus directly on raising the quality of instructional and socio-emotional interactions in such settings are sorely needed. Findings from this study confirm that TCIT-U positively predicts teachers’ sense of self-efficacy. Teachers with a higher sense of self-efficacy are more likely to effectively manage problem behaviors and are more committed to their role. Moreover, this study contributes to the knowledge of the effectiveness of the TCIT-U training program among Hispanic teachers, with varying levels of fluency in English, educational background, and early childhood teaching experience.

## Data Availability

The data are freely available from the corresponding author.
